# Impact of smoking, COPD and comorbidities on the mortality of COVID-19 patients

**DOI:** 10.1038/s41598-021-98749-4

**Published:** 2021-09-28

**Authors:** Donato Lacedonia, Giulia Scioscia, Carla Santomasi, Paolo Fuso, Giovanna Elisiana Carpagnano, Andrea Portacci, Franco Mastroianni, Giovanni Larizza, Eugenio Sabato, Emanuela Profilo, Emanuela Resta, Maria Pia Foschino Barbaro, Onofrio Resta

**Affiliations:** 1 Respiratory Diseases Unit, Department of Medical and Surgical Sciences, “Policlinico Riuniti” University Hospital, University of Foggia, Foggia, Italy; 2 Respiratory Diseases Unit, Cardio-Thoracic Department, “Policlinico di Bari” University Hospital, University of Bari “Aldo Moro”, Bari, Italy; 3 Internal Medicine COVID Unit, “F. Miulli” General Hospital, Acquaviva delle Fonti, 70021 Bari, Italy; 4 “Antonio Blasi” Respiratory Diseases Unit, “A. Perrino” P.O., 72100 Brindisi, Italy; 5 IRPPS-CNR, 00185 Rome, Italy; 6 Translational Medicine and Health System Management, University of Foggia, Foggia, Italy

**Keywords:** Microbiology, Pathogenesis, Risk factors, Signs and symptoms

## Abstract

The prognosis of the coronavirus disease 2019 (COVID-19) patients is variable and depends on several factors. Current data about the impact of chronic obstructive pulmonary disease (COPD) and smoking on the clinical course of COVID-19 are still controversial. This study evaluated the prevalence and the prognosis of COPD patients and smokers in a cohort of 521 patients admitted to four intermediate Respiratory Intensive Care Units (Puglia, Italy) with respiratory failure due to COVID-19 pneumonia. The prevalence of COPD and current smokers was 14% and 13%, respectively. COPD patients had a higher 30-day all-cause mortality than non-COPD patients. Former smokers compared to never smokers and current smokers had higher 30-day all-cause mortality. COPD patients and former smokers had more comorbidities. This study described the prevalence and the outcomes of COPD patients and smokers in a homogenous cohort of COVID-19 patients. The study showed that the prevalence of COPD and current smokers was not high, suggesting that they were not at increased risk of getting the infection. However, when SARS-CoV-2 infection occurred, COPD patients and former smokers were those with the highest all-cause mortality, which seemed to be mainly related to the presence of comorbidities and not to COPD and smoking itself.

## Introduction

The Severe Acute Respiratory Syndrome Coronavirus 2 (SARS-CoV-2) outbreak is an emerging health threat that has been declared a pandemic on March 11, 2020 by the Worth Health Organization (WHO)^1^. Coronavirus disease 2019 (COVID-19) has a broad spectrum of manifestations ranging from subclinical infection to critical illness. The mild disease has symptoms similar to usual upper respiratory tract infections^2,3^. In contrast, COVID-19 pneumonia is characterized by evidence of signs and symptoms (fever, and/or asthenia, and/or persistent cough) of lower respiratory disease during clinical evaluation and by presence of non-specific radiological figures at chest imaging (reticulations, and/or ground-glass, and/or consolidative opacities, predominantly distributed bilaterally and peripherally). It may lead to acute respiratory failure and evolve to acute respiratory distress syndrome, often requiring mechanical ventilation^4,5^.

Multiple studies investigate risk factors associated with a worse prognosis in COVID-19^6,7^. It has been noted that SARS-CoV-2 infection more readily causes respiratory failure and death in the most susceptible patients and in patients with comorbidities^8,9^. Chronic obstructive pulmonary disease (COPD) is a common chronic airway disease characterized by an irreversible impairment of lung functionality. Intuitively, since COVID-19 mainly affects the respiratory system, COPD may have high prevalence in COVID-19 population and may worsen clinical outcomes of these patients^10^. However, surprisingly, many studies showed that COPD was underrepresented among the comorbidities of COVID-19 patients compared with other comorbidities or with the global burden of the disease^11,12^. At the same time, COPD patients with COVID-19 had more severe disease than non-COPD patients^13^. Moreover, in June 2020 a WHO report warned that smoking could be a risk factor for the adverse prognosis of COVID-19^14^.

There are several available studies evaluating for associations between smoking status and SARS-COV-2 infection^15^. A recent systematic review did not report smoking as a risk factor for SARS-CoV-2 infection but reported an increased risk of severe disease and need for mechanical ventilation or death in current smokers^16^. On the other hand, another meta-analysis did not find an association between current smoking and the severity of the disease^17^. Therefore, the impact of underlying COPD and smoking on patients’ susceptibility and on the severity of SARS-CoV-2 infection is still uncertain and controversial^18,19^. In the present study, we hypothesized that COPD and tobacco smoking may have a different impact on patients’ susceptibility to SARS-CoV-2 infection and on the severity and clinical course of the disease. The primary aim was to analyse the prevalence of COPD and the prognosis of COPD patients in a selected and homogenous cohort of patients with acute respiratory failure due to COVID-19-related pneumonia and admitted to intermediate Respiratory Intensive Care Units (RICUs), a model of care designed for monitoring and treating respiratory patients whose illness is at a level of severity that is intermediate between that which requires intensive care unit (ICU) facilities and that which can be managed on a conventional ward^20,21^. The secondary aim was to examine the prevalence of smokers and the association of tobacco smoking with sociodemographic and clinical features during the clinical course of these hospitalized patients.

## Materials and methods

### Study design

This is a retrospective, observational and multicentric study conducted in four intermediate RICUs of the Puglia region in Southern Italy (Policlinico Riuniti of Foggia, Policlinico of Bari, “F. Miulli” Hospital of Acquaviva delle Fonti and “A. Perrino” Hospital of Brindisi). We obtained medical records and compiled data from hospitalized adult inpatients affected by COVID-19 related pneumonia and acute respiratory failure, that were managed from March 5th to May 31st, 2020. A total of 521 consecutive patients with SARS-CoV-2 pneumonia, confirmed by RT-PCR tests on nasopharyngeal swab and by chest X-ray or chest CT performed in Emergency Rooms, were included in the study. At admission, all patients underwent to routine blood examination, assessing the following laboratory parameters: complete blood count, coagulation profile with D-dimers, serum biochemical tests (including renal and liver function, albumin, cardiac enzymes and iron), inflammatory markers [C-reactive protein (CRP) and erythrocyte sedimentation rate], lactate dehydrogenase (LDH), total lymphocytes and T‐lymphocytes count, and vitamin D levels. The anamnesis, obtained both directly from the patients (if clinical conditions allowed) or from the relatives and/or clinical records, was aimed to ascertain smoking habits, the coexistence of respiratory diseases (such as asthma and COPD) and other comorbidities (diabetes mellitus, neurological diseases, kidney diseases, heart diseases, and cancer history). We used a semi-structured questionnaire in all the sites involved, investigating the same comorbidities. The anamnesis was also aimed at identifying patients on Long-term oxygen therapy (LTOT) or Long-term Non Invasive Ventilation (LTNIV) in order to assess the presence of chronic respiratory failure at baseline. Age-corrected Charlson Comorbidity Index (CCI) was calculated. The required ventilatory support and the arterial oxygen partial pressure to fractional inspired oxygen ratio (PaO_2_/FiO_2_) ratio of each patient at admission were recorded. Since deaths occurred in hospital, the death data was obtained from the hospital databases. Data on 30 day-survival were also collected. On the basis of anamnestic data, the enrolled subjects were then divided into 5 groups to assess differences: (1) Current smokers with COPD, (2) Former smokers with COPD, (3) Current smokers without COPD, (4) Former smokers without COPD and (5) Never smokers. In the former smokers groups, we included those who had a history of smoking tobacco cigarettes in the past for at least 1 year and had stopped smoking for at least 3 months.

All patients’ data were collected in the context of routine clinical care, and written informed consent was signed at admission according to hospital policy. Ethical Committee “Area 1 dell’Azienda Ospedaliero-Universitaria, Ospedale Riuniti” of Foggia approved this study. The experiment was performed in accordance with relevant guidelines and regulations.

### Statistical analysis

Continuous variables are presented as arithmetic mean ± standard deviation (SD), while categorical variables as counts (N) and percentage (%). Student T-test and ANOVA (with Turkey post-Hoc analysis) were used for comparing continuous data. Chi-square test (χ^2^) was used to assess the relationship between categorical variables. The Kaplan–Meier method with hazard ratio (HR) was applied to detect differences in survival between the groups. A Cox proportional hazard model was used to estimate adjusted HRs with 95% confidence intervals (CIs) and to assess the influence of age, investigated comorbidities, CCI, and smoking habits. The cut off of the CCI was identified by a receiving operator curve (ROC) analysis, and a value greater than or equal to 4 was found to be the one with greater specificity (63%) and sensitivity (85%). A p value of < 0.05 was considered to be significant.

## Results

A total of 521 COVID-19 patients were enrolled. Baseline characteristics of whole population are shown in Table 1. Data on smoking habits was available in 507 patients. According to a history of COPD and smoking, patients were divided into 5 groups (Table 2).

**Table 1:** Sociodemographic and clinical characteristics of the whole population. i.e., 521 COVID-19 patients hospitalized in four intermediate Respiratory Intensive Care Units (RICUs).

N	521	
N without smoking information	14	
	Mean	SD
Age	67.18	17.57
Sex M%	53%	
Smoking habits (Never—Current—Former) %	55%—13%—32%	
Asthma %	1%	
COPD %	14%	
Diabetes mellitus %	17%	
Neurological diseases %	11%	
Heart diseases %	17%	
History of cancer %	11%	
Kidney diseases %	25%	
30-day all-cause mortality %	25%	
CCI	3.35	2.322
Lymphocytes	1126.79	699.024
PaO_2_/FiO_2_	250.92	108.555
D-Dimer	2677.36	6221.999
CRP	51.24	72.585
LDH	328.22	205.241

*SD* standard deviation, *COPD* chronic obstructive pulmonary disease, *PaO*_*2*_*/FiO*_*2*_ arterial oxygen partial pressure to fractional inspired oxygen ratio, *CCI* Charlson Comorbidity Index, *CRP* C-reactive protein, *LDH* lactate dehydrogenase.

**Table 2:** Sociodemographic and clinical characteristics of subjects according to the smoking habits and the presence of COPD (chronic obstructive pulmonary disease).

	CS + COPD		FS + COPD		CS–NO COPD		FS–NO COPD		NS–NO COPD		p
N (%)	13 (2.7%)		57 (11.2%)		55 (10.8%)		105 (20.7%)		275 (54.2%)		
	Mean	SD	Mean	SD	Mean	SD	Mean	SD	Mean	SD	
Age	72.44	18.52	79.53	12.04	58.30	15.67	66.90	18.68	66.12	17.23	< 0.01
Sex (M) N (%)	11 (85%)		27 (47%)		43 (78%)		50 (48%)		137 (50%)		< 0.01
Asthma N (%)	0 (0%)		0 (0%)		0 (0%)		0 (0%)		5 (2%)		0.40
COPD N (%)	13 (100%)		57 (100%)		0 (0%)		0 (0%)		0 (0%)		
Diabetes mellitus N (%)	5 (38%)		5 (9%)		8 (15%)		12 (11%)		52 (19%)		< 0.05
Neurological diseases N (%)	3 (23%)		10 (18%)		4 (7%)		8 (8%)		30 (11%)		0.23
History of cancer N (%)	3 (23%)		15 (26%)		2 (4%)		6 (6%)		30 (11%)		< 0.01
Heart diseases N (%)	4 (31%)		22 (39%)		6 (11%)		10 (10%)		44 (16%)		< 0.01
Kidney diseases N (%)	5 (38%)		26 (46%)		5 (9%)		37 (35%)		52 (19%)		< 0.01
CCI	5.62	2.06	6.02	1.63	2.00	1.99	3.13	2.14	3.05	2.13	< 0.01
30-day all-cause mortality %	54%		54%		5%		39%		17%		< 0.01
Lymphocytes	1002.87	708.23	947.58	694.07	1225.75	609.36	1350.73	956.60	1064.86	570.85	< 0.01
PaO_2_/FiO_2_	250.77	156.06	222.22	89.14	265.40	115.52	249.24	112.77	256.48	106.08	0.21
CRP	67.77	77.13	57.00	71.67	56.72	73.18	47.08	70.12	46.58	71.80	0.09
D-Dimer	7188.46	14,594.05	2419.83	3673.34	3141.48	9768.87	2970.79	5618.29	2142.24	4828.50	0.65
LDH	294.00	92.61	358.12	155.98	275.94	115.03	302.51	127.56	344.70	253.08	0.15

*SD* standard deviation, *CS* smoker, *FS* former smoker, *NS* never smoker, *PaO*_*2*_*/FiO*_*2*_ arterial oxygen partial pressure to fractional inspired oxygen ratio, *CCI* Charlson Comorbidity Index, *CRP* C-reactive protein, *LDH* lactate dehydrogenase.

Only 2 patients had COPD and had never smoked, so they were not included in the table.

COPD patients were 72 (14%) and most of them were current or former smokers (97.2% of current and former smokers vs. 2.8% of never smokers). Current smokers with COPD were mainly males (85%), while in the group of former smokers with COPD, male and female patients were similarly represented. Mean age of COPD patients was 78.150 ± 13.541. Main comorbidities were kidney diseases (44%), heart diseases (36%), history of cancer (25%), neurological diseases (18%) and diabetes mellitus (15%). At admission, mean PaO_2_/FiO_2_ ratio of COPD patients was 231.67 ± 106.40, mean CRP was 57.46 ± 71.84, mean D-dimer 3367.60 ± 7489.84, mean LDH 341.79 ± 147.52, number of peripheral lymphocyte cells 953.42 ± 680.83.

Current smokers without COPD were 55 (10.8%), mainly males (78%), and their mean age was 58.30 ± 15.67. The main comorbidities of current smokers without COPD was diabetes mellitus (15%), followed by heart diseases (11%), kidney diseases (9%), neurological diseases (7%) and history of cancer (4%). At admission, the mean PaO_2_/FiO_2_ ratio of these patients was 265.40 ± 115.52, mean CRP was 56.72 ± 73.18, mean D-dimer 3141.48 ± 9768.87, mean LDH 275.94 ± 115.03, number of peripheral lymphocyte cells 12,225.75 ± 609.36.

In the group of former smokers without COPD (105 patients, 20.7%), male and female patients were similarly represented (males 48% vs. females 52%). Mean age was 66.90 ± 18.68. A lot of them had comorbidities: 35% kidney diseases, 11% diabetes mellitus, 10% heart diseases, 8% neurological diseases and 6% history of cancer. At admission, the mean PaO_2_/FiO_2_ ratio of this group was 249.24 ± 112.77, mean CRP was 47.08 ± 70.12, mean D-dimer 2970.79 ± 5618.29, mean LDH 302.51 ± 127.56, number of peripheral lymphocyte cells 1350.73 ± 956.60.

Never smokers without COPD were 275 (54.2%). In this group, the two sexes were equally represented. Mean age was 66.12 ± 17.3. The comorbidities were: diabetes mellitus (19%), kidney diseases (19%), heart diseases (16%), neurological diseases (11%) and history of cancer (11%). At admission, the mean PaO_2_/FiO_2_ ratio was 256.48 ± 106.08, CRP 46.58 ± 71.80, D-dimer 2142.24 ± 4828.50, LDH 344.70 ± 253.08 and the mean number of peripheral lymphocyte cells was 1064.86 ± 570.85.

Out of 521 subjects, five required LTOT and none required LTNIV at baseline. Of the patients requiring LTOT, 2 had COPD (2.7%) and 3 were never-smokers without COPD.

**Figure 1:**
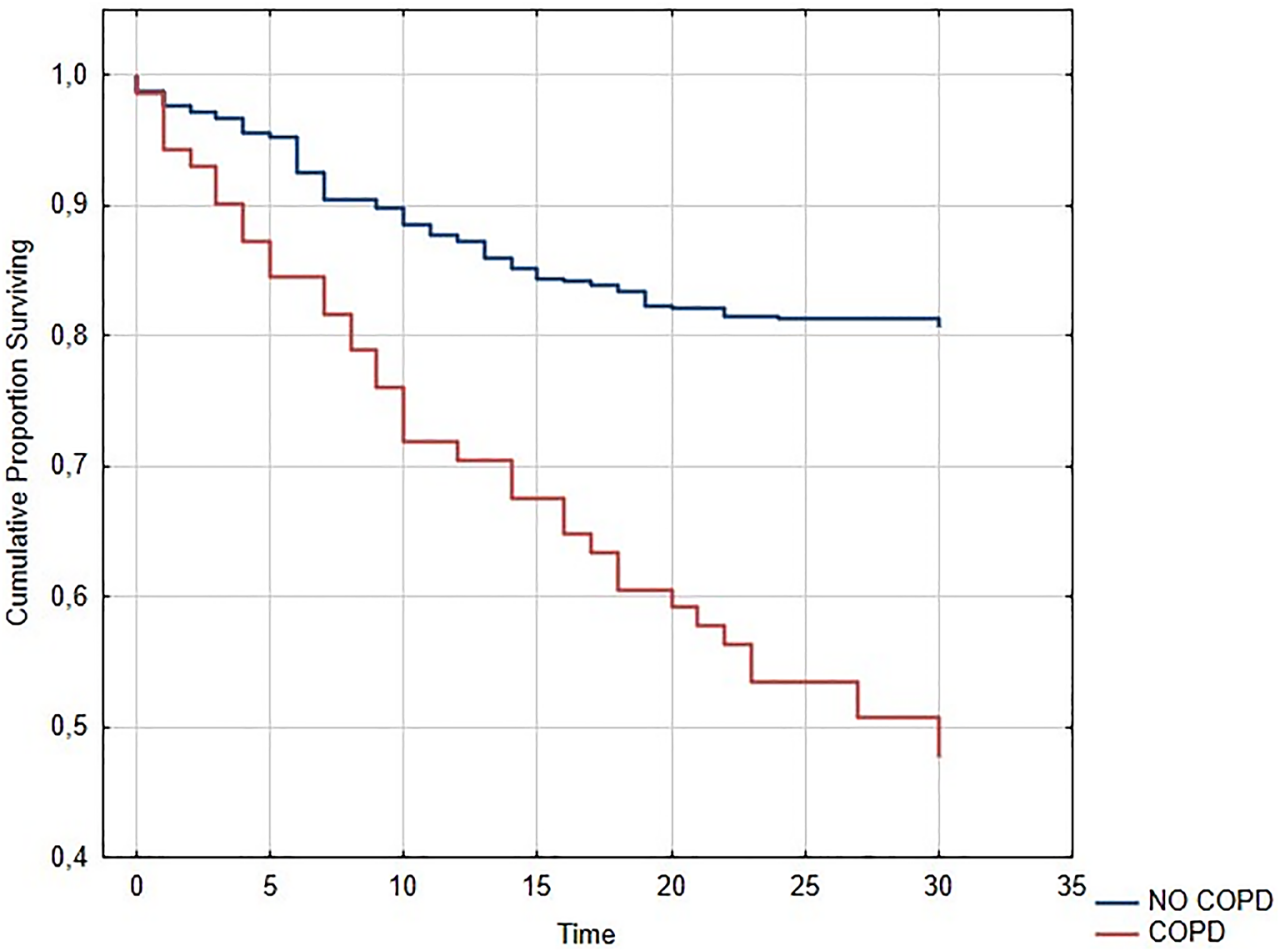
Kaplan–Meier plot of the probability of survival in COVID-19 patients hospitalized in four intermediate Respiratory Intensive Care Units according to the presence of chronic obstructive pulmonary disease (COPD).

**Figure 2:**
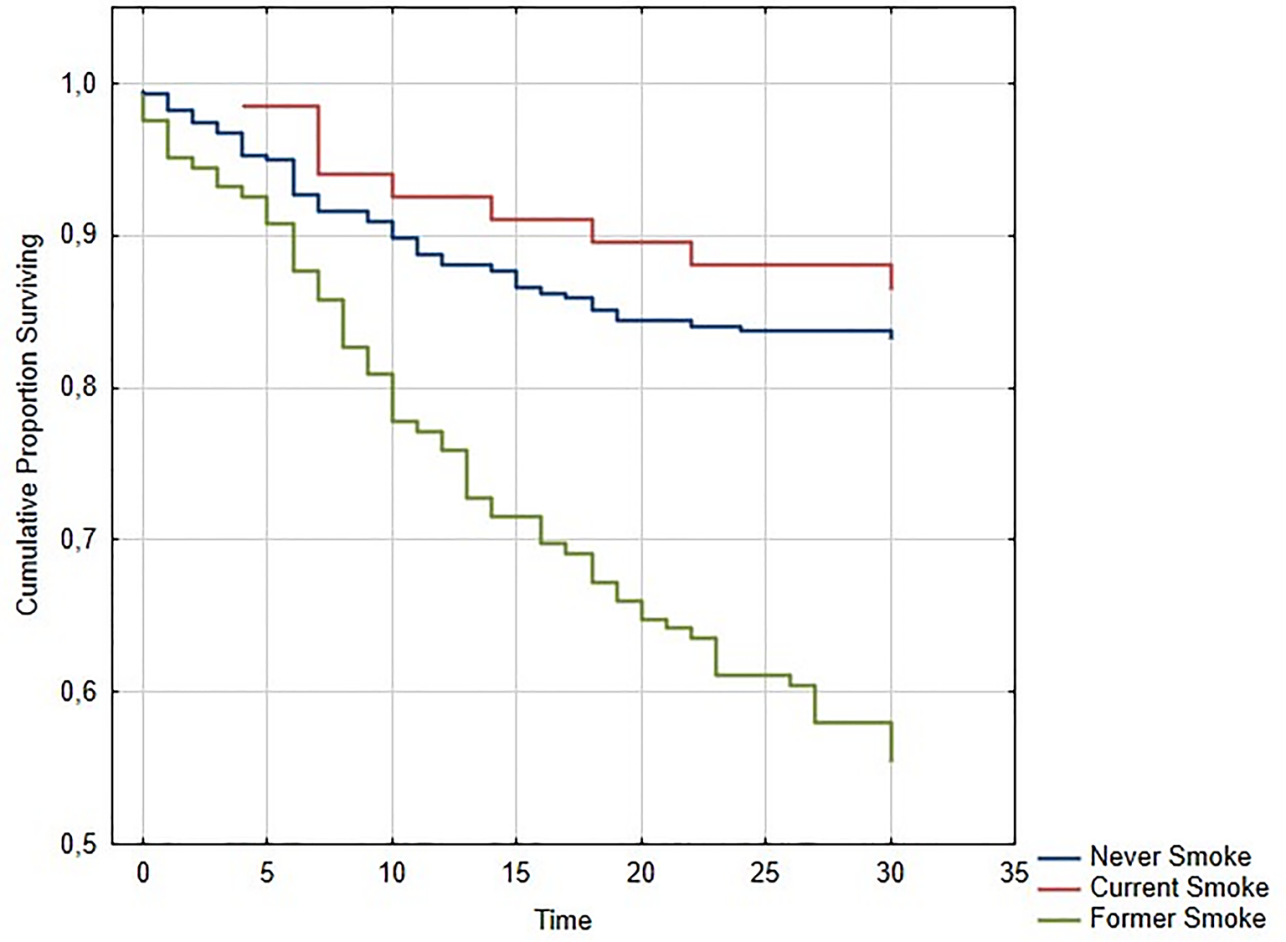
Kaplan–Meier plot of the probability of survival in COVID-19 patients hospitalized in four intermediate Respiratory Intensive Care Units according to smoking habits.

COVID-19 patients with COPD were significantly older than COVID-19 patients without COPD (COPD mean age: 78.15 ± 13.54 vs. non COPD: 65.66 ± 16.85; p < 0.0001). The CCI calculated for COPD patients was significantly higher than the one of COVID-19 patients without COPD (COPD: mean CCI 5.92 ± 1.73 vs. non-COPD: mean CCI 2.94 ± 2.14; p = 0.001). COPD patients had also a 30-day all-cause mortality significantly higher than non-COPD patients (52% vs. 21%, p < 0.0001—Fig. 1). Statistical analysis performed on the population of smokers with and without COPD revealed that former smokers had higher 30-day all-cause mortality compared to never smokers and current smokers (former smokers: 44%, current smokers: 13%, and never smokers: 17%; p < 0.0001—Fig. 2). Current smokers had lower 30-day mortality than former smokers, but they were younger (mean age of never smokers: 66.10 ± 17.22 years; mean age of current smokers: 60.69 ± 16.98 years; mean age of former smokers: 71.41 ± 17.64 years; p = 0.0001). Former smokers had also a significantly higher CCI than current and never smokers (mean CCI of former smokers: 4.16 ± 2.41; mean CCI of never smokers: 3.07 ± 2.13; mean CCI of current smokers: 2.64 ± 2.44; p = 0.0001).

Data on the ventilatory support required at admission were available only for 256 COVID-19 patients (Table 3). In this regard, the COPD group was significantly associated with the need to use mechanical ventilation in bilevel positive airway pressure (BPAP) mode [37 (16.97%) non-COPD patients vs*.* 15 (39.47%) of COPD patients underwent BPAP mode; p = 0.001].

**Table 3:** Differences in ventilatory support of COVID-19 patients hospitalized in four intermediate Respiratory Intensive Care Units (RICUs) according to the presence of chronic obstructive pulmonary disease (COPD) (N = 256).

Group	Total	AA	LFOT	HFNC	CPAP	BPAP
Without COPD	218 (85.10%)	31 (14.22%)	75 (34.40%)	6 (2.75%)	69 (31.65%)	37 (16.97%)
With COPD	38 (14.84%)	0 (0.00%)	10 (26.32%)	1 (2.63%)	12 (31.58%)	15 (39.47%)
p		0.01	> 0.05	> 0.05	> 0.05	0.001

*AA* ambient air, *LFOT* low-flow oxygen therapy, *HFOT* high-flow oxygen therapy, *CPAP* continuous positive airway pressure, *BPAP* bilevel positive airway pressure.

The univariate Cox proportional hazard analysis of the 30-day relative risk of all-cause mortality on the whole population showed that age ≥ 65 years, smoking habits, CCI ≥ 4 and the presence of the different comorbidities investigated (except of history of cancer) increased all-cause mortality. However, after multivariate analysis, only age, smoking habits and some comorbidities (heart and neurological diseases) confirmed to be the greater risk factors of all-cause mortality, but not COPD (smoking habits: HR: 1.68, 95% CI: 1.34–2.11; age ≥ 65 years: HR: 3.48, 95% CI: 2.26–5.37; neurological diseases: HR: 2.37, 95% CI: 1.56–3.60; heart diseases: HR: 1.75, 95% CI: 1.16–2.64)] (Table 4).

**Table 4:** Univariate Cox proportional hazard model and multivariate Cox proportional hazard model of the 30-day relative risk of all-cause mortality in COVID-19 patients hospitalized in four intermediate Respiratory Intensive Care Units (RICUs).

	Univariate analysis				Multivariate analysis			
	HR	95% CI Lower	95% CI Higher	P	HR	95% CI Lower	95% CI Higher	P
Age ≥ 65 years	5.80	3.93	8.58	< 0.01	3.48	2.26	5.37	< 0.01
Gender	0.69	0.49	0.97	< 0.05	0.80	0.56	1.14	
Smoking	1.78	1.47	2.16	< 0.01	1.68	1.34	2.11	< 0.01
COPD	2.92	2.00	4.27	< 0.01	1.00	0.63	1.60	
Neurological diseases	4.21	2.85	6.23	< 0.01	2.37	1.56	3.60	< 0.01
Diabetes mellitus	1.57	1.04	2.37	< 0.05	1.53	1.00	2.36	0.051
Heart diseases	3.18	2.21	4.57	< 0.01	1.75	1.16	2.64	< 0.01
History of cancer	1.16	0.70	1.90	0.56				
Kidney diseases	3.27	2.32	4.61	< 0.01	1.47	1.00	2.17	0.051
CCI ≥ 4	7.53	4.67	12.12	< 0.01				

*HR* hazard ratio, *CI* confidence interval, *COPD* chronic obstructive pulmonary disease, *CCI* Charlson Comorbidity Index.

Multivariate hazard ratio included age, gender, smoking, COPD, neurological, kidney and heart diseases.

## Discussion

To the best of our knowledge, this retrospective observational multicentre study was the first study that described the prevalence of COPD and the prognosis of COPD patients in a selected and homogeneous cohort of patients admitted to intermediate RICUs with COVID-19-related pneumonia. The present study also described the prevalence of smokers and the association of tobacco smoking with sociodemographic and clinical features during the clinical course of these hospitalized patients.

One of the most notable findings in our study was the higher prevalence of COPD (14%) in COVID-19 population compared with previous reports. In this regard, Attaway and colleagues showed that in a COVID-19 symptomatic population of 15.586 patients, there were no significant differences in the rate of SARS-COv-2 positivity between COPD and non-COPD patients^22^. However, the study itself showed a higher prevalence of COPD patients that required hospitalization (48.2% of COPD population vs. 26.5% of non-COPD population)^22^. Other studies reported a COPD prevalence of 2% in COVID-19 patients^23^. Emami and colleagues showed a COPD incidence of 0.95% in hospitalized COVID-19 patients^24^. The Spain registry of hospitalized patients with COVID-19 showed a relatively low COPD incidence of 7.7%^25^. Our previous study conducted on 97 hospitalized COVID-19 patients also showed a low percentage of respiratory comorbidities (8.3%) and therefore of COPD^12^. To date, only the review article of Leung and colleagues has suggested an extremely variable prevalence (from 1.2 to 38%) of COVID-19 patients with COPD, which is the closest to the present finding^26^. A possible explanation of our finding concerns the higher age of our population (mean age 67.18 ± 17.5 years). One another possible explaining hypothesis is that reduced use of drugs for respiratory diseases, such as inhaled corticosteroids or bronchodilators, might have favoured the infection. In fact, corticosteroids could represent a protective factor against COVID-19^22^. Another possible hypothesis concerns the greater disease severity of our population, with patients with COVID-19-related pneumonia and acute respiratory failure. COVID-19 patients with COPD, being at greater risk of the severe forms of the disease, could be more frequent in intermediate RICUs. Finally, since our definition of COPD was based only on clinical history and not on spirometric values, this might have led to COPD overdiagnosis.

Whether COPD could represent a risk factor for negative prognosis in COVID-19 remains to be clarified. However, when we talk about evidence in this area, we must consider that the race to publish, especially in the first phase of the pandemic, may have led to publication of studies with a lot of methodological biases (e.g., poor statistical analysis, non-standardized data collection methods, non-homogeneous populations, too small simple sizes, unadjusted prevalence data) and to erroneous conclusions, which may be responsible for current controversies. In our study, COPD was associated to a major requirement of non-invasive ventilation in BPAP mode (16.97% of non-COPD patients vs. 39.47% of COPD patients underwent BPAP mode; p = 0.001) and to a higher 30-day all-cause mortality (non-COPD patients: 21% vs. COPD patients: 52%; p = 0.0001). These findings are in agreement with those of Attaway and colleagues showing that higher rates of ICU admission [adjusted odds ratio (OR): 1.20) and invasive mechanical ventilation (adjusted OR: 1.49) were observed in COPD patients compared with non-COPD patients in a COVID-19 population^22^. Guan and colleagues described similar findings, as they showed that COPD patients were more likely to experience ICU admission, invasive ventilation, and death^8^. Taken together, these data confirmed that COPD could be a risk factor for severe COVID-19. But, how can COPD be responsible for more severe forms of COVID-19? An intriguing possibility concerns the ACE-2 enzyme, which is essential for the virus to gain entry into the organism and it is overexpressed in the lower respiratory tract of COPD patients, favouring the severity of the disease^27^. Another hypothetical explanation is the alteration of the renin–angiotensin–aldosterone system in COPD patients, which could lead to pulmonary edema during SARS-CoV-2 infection^28^. The presence of patients with severe forms of COPD at baseline might be a further explanation. In particular, in patients with severe COPD without COVID-19 the presence of chronic respiratory failure and, therefore, the use of LTOT, is associated with negative consequences which might also be responsible for the poor prognosis of patients with severe COPD and COVID-19^29,30^. However in our population only 2 (2.7%) COPD patients with COVID-19 had a history of LTOT/chronic respiratory failure, not explaining the worst prognosis of these patients. By contrast, in the present study, the higher age and the presence of comorbidities in COPD patients may explain the greater severity of their disease. In fact, while the univariate analysis on our sample showed that age, smoking, individual comorbidities and CCI ≥ 4 increase the 30-day relative risk of all-cause mortality, the multivariate analysis revealed that it was not COPD itself the cause of the greater severity of COVID 19, but are the higher age, the smoking habits, and the presence of other comorbidities (in particular cardiovascular and neurological ones). Since CCI is an index that includes both age and comorbidities, it could represent a valid tool for predicting the prognosis of these patients. These findings are in agreement with those of Gómez Antúnez and colleagues obtained from a study conducted on 10,420 patients^13^. In particular, the association between multimorbidity and negative prognosis of COPD patients had already been widely emphasized by Almagro and colleagues in the field of COPD exacerbation. Their most recent studies suggested that 3-month mortality in hospitalized COPD patients was correlated to a higher CCI^31,32^. Therefore, the increased presence of multimorbidity may be responsible for the worse prognosis in COVID-19 patients affected by COPD.

Another important point of discussion of the present study was to understand whether current smokers are more prone to contract SARS-CoV-2 infection. Since several studies in the past have shown that smokers are predisposed to severe respiratory infections, it would be logical to expect a high prevalence of current smokers in hospitalized COVID-19 patients^33^. Oppositely, in our population of patients hospitalized with COVID-19 pneumonia, we found a prevalence of current smokers of 13%, which is low if compared with the 20.3% prevalence of smokers in the 60–64 age class and with the 14.4% prevalence in the 65–74 age class of the general Italian population^34^. This surprising and paradoxical finding is in line with most similar studies. In particular, a Chinese analysis conducted on hospitalized COVID-19 patients showed a low prevalence of smokers (6.5%) compared to that of the general Chinese population^35^. Miyara and colleagues revealed a low incidence of current smokers even in a COVID-19 French population^36^. On the contrary, Guan and colleagues found a higher prevalence of current smokers in severe forms of the disease than in milder ones, suggesting that the prevalence may vary depending on the severity of the examined population^6^. In this regard, we would like to point out that, although our population was mostly made up of severe patients with acute respiratory failure, the prevalence of current smokers remained low. Also Cumming and colleagues reported a low prevalence of smokers in critically ill patients with COVID-19^37^. The low rate of smokers observed in COVID-19 patients suggests that smokers are less prone to contract SARS-CoV-2 infection and that smoking habit has a “protective” effect. Several mechanisms may explain how this "smoker's paradox" may be possible^38,39^. A possible mechanism concerns the nitric oxide produced during smoking, which inhibits the replication of the virus and its entry into cells. Another explanation applies the reduced expression of ACE-2 in smokers, which could reduce the risk of attachment of the virus to upper respiratory tract cells and the consequent infection^19^. However, the reduced expression of ACE-2 itself may be harmful, as it exposes the smoker to a greater risk of local inflammation, vasoconstriction and thrombosis worsening his outcome^19^.

In fact, about the link between smoking and clinical outcomes of COVID-19, a systematic review concluded that smoking was associated with disease progression and increased adverse outcomes in COVID-19^16^. Similar findings derived from a very large study of the UK general population, where smoking was significantly associated with increased COVID-19 mortality after age and sex adjustment (OR: 1.25, 95% CI: 1.12–1.40). However, after adjustment for multiple additional covariates, the same study found that smoking was associated with a reduced risk for COVID-19 mortality (OR: 0.88, 95% CI: 0.79–0.99)^40^. Moreover, other studies in particular analysed the relative risk of current smokers. Lippi and Henry’s meta-analysis demonstrated no significant association between current smoking and COVID-19 severity (OR: 1.69, 95% CI: 0.41–6.92; p = 0.254)^17^. On the other hand, some reviews have highlighted an increased mortality and need for mechanical ventilation in current smokers^15^. Due to these controversies, different mechanisms by which smoking could exert a protective or harmful action on the clinical course of COVID-19 have been proposed. A possible protective mechanism of smoking relates to the inhibition of pro-inflammatory cytokines production by nicotine, protecting against cytokine-storm syndrome. Another theory concerns the continuous suppression of these systemic cytokines in smokers, who may adapt their immune response by becoming more tolerant and less reactive to continuous inflammatory stimuli than patients who have never smoked. However, smokers have a higher prevalence of comorbidities, that are known to have an independent negative impact on the clinical course of COVID-19^19^. A very important and innovative finding of the present study was that, among smokers, former smokers were those with higher mortality. Since former smokers had higher multimorbidity than never smokers and current smokers, and since the multivariate analysis showed comorbidities like neurological and cardiovascular ones among the risk factors for mortality, we can hypothesize that it was not smoking itself the responsible for the worst prognosis of smokers, but the comorbidities related to its chronic exposure.

The present study had some limitations. Firstly, since patients were often critically ill at admission, pulmonary function tests could not be performed. Therefore, the diagnosis of COPD was based on purely anamnestic data. Secondly, we analysed data basing on what was available in the hospital databases, but we recognized there were some information not captured by them, including knowledge about existing medications, through which we would have confirmed or not the diagnosis of COPD. Furthermore, we did not have cause-specific data on mortality. Moreover, given that the present study analyzed outcomes from March 5th to May 31st 2020, therefore before corticosteroids were routinely used, the lack of this medication in the first phase of the pandemic may have influenced all-cause mortality in our hospitalized COVID-19 patients. Finally, we did not have a cumulative effect of smoking over time, as measured by pack-years. In fact some studies have suggested that the cumulative exposure to cigarette smoking was an independent risk factor for hospital admission and death from COVID-19^41^.

## Conclusions

Based on our study findings, in a selected and homogeneous cohort of hospitalized COVID-19 patients, the prevalence of COPD was not high, suggesting that they were not at increased risk of getting the infection. However, when SARS-CoV-2 infection occurred in COPD patients, they had a higher all-cause mortality and a worse prognosis than non-COPD patients. The present study also showed that SARS-CoV-2 infection was not common in current smokers and that, among smokers with COVID-19, former are those with the higher mortality. The worst outcomes of COPD patients and former smokers seemed to be mainly related to the comorbidities of these subjects and not to COPD or smoking itself.
